# Psoriatic Arthritis: Development, Detection and Prevention: A Scoping Review

**DOI:** 10.3390/jcm12113850

**Published:** 2023-06-05

**Authors:** Agnieszka Kimak, Ewa Robak, Joanna Makowska, Anna Woźniacka

**Affiliations:** 1 Department of Dermatology and Venereology, Medical University of Lodz, Hallera 1, 90-647 Lodz, Poland; ewa.robak@umed.lodz.pl (E.R.); anna.wozniacka@umed.lodz.pl (A.W.); 2 Department of Rheumatology, Medical University of Lodz, Zeromskiego 113, 90-549 Lodz, Poland; joanna.makowska@umed.lodz.pl

**Keywords:** development, early intervention, prevention, psoriasis, psoriatic arthritis, ultrasonography

## Abstract

Psoriatic arthritis is a heterogenous chronic inflammatory disease that develops over time in some patients with psoriasis. The course of the disease is variable, with a broad clinical spectrum. The management of PsA has changed tremendously over the last decade, thanks to earlier diagnosis, a multidisciplinary approach and progress in pharmacological therapies. Therefore, screening for risk factors and the early signs of arthritis is highly important and recommended. Currently, research is focused on finding soluble biomarkers and developing imaging techniques that can improve the prediction of psoriatic arthritis. Among imaging modalities, ultrasonography seems to be the most accurate in detecting subclinical inflammation. Early intervention is based on the assumption that it is possible to prevent or delay psoriatic arthritis if systemic treatment for psoriasis can be administered early enough. This review article provides an overview of the current perspectives and evidence regarding the diagnosis, management and prevention of psoriatic arthritis.

## 1. Introduction

Although psoriasis (PsO) and psoriatic arthritis (PsA) are closely associated, the relationship between these conditions is not fully understood. While it has been established that some groups of psoriasis patients are predisposed to arthritis, many patients are still diagnosed in the late stages of PsA, when lesions are irreversible. Therefore, the present paper reviews the existing evidence regarding the screening for risk factors and early signs of arthritis, and the possible influence of early medical intervention, on the development of PsA. A literature search was conducted on the PubMed MEDLINE database for articles in English using the following search terms: (psoriasis) AND (psoriatic OR arthritis OR prevention OR development). Publications from 2010 through March 2023 were scrutinized. Additional relevant publications were obtained by analyzing the reference sections from chosen articles.

## 2. Risk Factors for PsA in Psoriasis Patients

Psoriatic arthritis is a form of chronic inflammatory arthritis that is prevalent in one in five patients with PsO [1]. Of these patients, 30% develop joint inflammation without preceding skin lesions [2]. PsA is traditionally divided into five types based on clinical assessment: distal interphalangeal joint arthritis, asymmetrical oligoarthritis, polyarthritis, spondylitis and arthritis mutilans [3]. However, as inflammation occurs as a dynamic process in PsA, the number of involved joints and clinical patterns may change over time, making the classification unreliable [4]. The most common PsA manifestation is oligoarthritis (13–26%) and polyarthritis (59–68%), and a progression from oligo- to polyarthritis is often observed. Apart from its traditional division, dactylitis and enthesitis are the most prevalent forms [5].

Several factors have been shown to increase the risk of PsA development, while a few remain controversial. Regarding the skin lesion severity threshold, PASI (Psoriasis Area and Severity Index) > 20, >10 or even >6 have been noted as risk factors for arthritis development; a recent prospective population-based cohort study found the hazard ratio for PsA development to be 1.44 in psoriasis patients with BSA (Body Surface Area) 3–10% and 2.01 for those with BSA > 10% [2,6,7,8]. The risk of PsA development was also influenced by location: the risk was twofold greater in the intergluteal area, three times greater in the nail area, and four times greater on the scalp [9]. In 2019, the nail expert group consensus acknowledged an association between nail psoriasis and the increased risk of PsA and recommended systemic treatment with conventional or biological agents in patients with more than three nails affected by PsO regardless of skin involvement [10].

Psoriatic arthritis has a genetic background, and as much as 40% of PsA patients have a positive family history of PsO and PsA. These patients have an earlier onset of the disease, more frequent nail involvement, enthesitis and deformities, and less often achieve minimal disease activity [11,12]. An HLAB27 haplotype and nucleotide polymorphism in the IL-17, IL-23 and NF-κB axis have been associated with a higher risk of PsA development. On the other hand, the HLA-Cw*06 haplotype, which is strongly associated with psoriasis, was found in less than 30% of PsA patients. Epigenetic factors may also play a role, with IL22 hypermethylation suggested to be a possible germ-line risk locus for PsA [13]. Another study found PsO and PsA patients differed with regard to DNA methylation patterns in CD8+ T cells in the blood, and this correlated with disease activity [14].

Abdominal obesity and BMI have been found to influence psoriatic arthritis; this has been attributed to an increase in the level of inflammatory cytokines associated with adipose tissue and the functional overload of entheses, especially in the lower legs, leading to microtraumas that initiate an inflammatory response [2,6,15]. Psoriasis is associated with metabolic syndrome, and PsO patients are more likely to demonstrate obesity, hypertension, hyperlipidemia, type 2 diabetes and cardiovascular disease. Psoriasis management, therefore, requires a multidisciplinary approach based on introducing preventive measurements and treating comorbidities [15,16].

It is unclear whether smoking is an additional PsA risk factor; in addition, no association has been found with alcohol consumption, and the role of gut microbiota remains unclear [2]. Although research studies are lacking, the PsA population appears to demonstrate a higher prevalence of autoimmune conditions, such as vitiligo, systemic lupus erythematosus, celiac and thyroid disease compared to the general population [17].

Some of the discussed characteristics may not be useful in psoriasis patients receiving biological treatment. Van Muijen et al. found that 9.4% of psoriasis patients receiving biological treatment to develop PsA, and more importantly, 53.8% of these patients had PASI < 5 at the moment of PsA diagnosis. The authors concluded that known clinical risk factors might be inaccurate for predicting the onset of PsA in patients with moderate-to-severe psoriasis treated with biologics; furthermore, even with low disease activity, these patients can progress to PsA [18].

## 3. Psoriatic Arthritis Development

**Figure 1:**
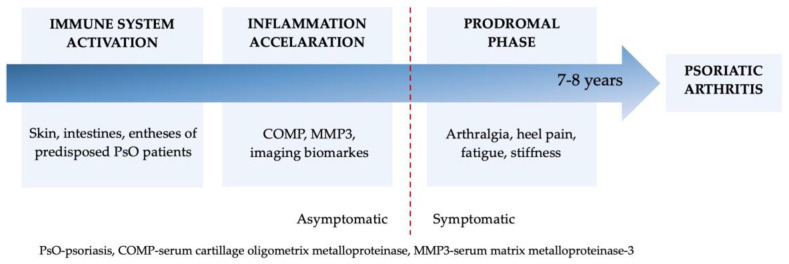
Possible transition from psoriasis to psoriatic arthritis.

The development of psoriatic arthritis typically takes seven to eight years, during which three reversible stages can be differentiated [19] (Figure 1).

In patients predisposed to psoriatic arthritis, the initial phase is characterized by a pre–clinical immune system activation in the skin, intestinal musosa or entheses. The immune system can be activated by a mechanical trigger, as demonstrated in mouse [20] and human models [21]. McGonagle proposes that enthesis inflammation may serve as an immunopathogenic basis for PsA [22]. In the second phase, the inflammation accelerates, and specific cellular, tissue, soluble and imaging biomarkers can be detected in patients who remain asymptomatic. Finally, in the third stage, also called prodromal PsA or psoriatic inflammatory arthralgia, non-specific musculoskeletal symptoms, such as arthralgia, heel pain, fatigue and stiffness, could be observed; these were not explained by other diagnoses and typically occurred months before any detection of synovitis or enthesitis during a physical examination [6,23].

Although several classification criteria have been proposed for diagnosing PsA, most require a diagnosis of true arthritis. However, the CASPAR criteria permit the diagnosis of psoriatic arthritis in patients with dactylitis and enthesitis without joint or spine involvement. This classification is the preferred tool when detecting the early stages of PsA, with a sensitivity of 0.914 and a specificity of 0.987 [24].

## 4. Genetic and Pharmacogenetic Biomarkers

The distribution of human leukocyte antigen (HLA) classes, genetic polymorphisms and micro-RNA expression could potentially serve as PsA-specific genetic and pharmacogenetic markers. For example, PsA development was found to be correlated with HLA-B*27, HLA-B*38, HLA-B*39, HLA-C*12 and HLA-C*06 in the European population [25,26], and HLA-A*01 in Chinese Han population [27]. Moreover, several non-HLA loci, such as Single Nuclear Polymorphisms (SNPs) in the *IL-13* gene [28], *VEGF* gene [29] and *IL-33* gene [30], were confirmed to be associated with PsA. Multiple gene regions required further investigation. In one analysis, in which SNPs mapping 44 genomic regions was undertaken, evidence was found for 5q33.3, 1p36, 1q21.3 loci [31]. In addition, MiR-21-5p upregulation in one of the mi-RNA signatures was identified in patients with active PsA and possibly might be used in the monitoring of response to treatment [32]. A similar role was suggested for miR-941, which was found in CD14+ monocytes [33].

Pharmacogenetic biomarkers facilitate the identification of genetic polymorphisms that are associated with variability in interindividual drug response. Although the results of PsA studies are often contrasting, several described polymorphisms are worth mentioning [34]. For example, according to Ando et al., the SNPs in the *reduced folate carrier 1 (RFC1)* gene correlate with a higher concentration of the active metabolite of methotrexate (MTX) in erythrocytes [35]. Variations in efflux transporter genes, such as *ABCC1* and *ABCG2*, correlate with a positive outcome to MTX [36]. The differences in the frequency of HLA-Cw6, *FOXP3* and *ANxA6* were also found between MTX responders and non-responders [37,38]. In addition, MTX toxicity was more prevalent in carriers of *TYMS 50-UTR 3R* and *SLC19A1* polymorphisms [37]. Data for other conventional drugs that are used in PsA treatment is rather scant and inconclusive. In terms of acitren, the rs2282143CT–rs4149056TC haplotype was correlated with better outcomes [39]. Similar results were observed for the Val/Val GSTP1 polymorphism in dimethyl fumarate users [40]. The investigation on biomarkers of biological efficacy is unfortunately lacking and not significant. HLA-Cw*06:02 seems to have a poor prognostic value [34]. A few SNPs, such as those located on *IL28RA, VEGFA, CYLD, LMO4, IL12B, TNFAIP3, TLR10, MICA-A9, TRAF3IP2* and *SDC4* genes for adalimumab or rs35569429 on chromosome 4 for ustekinumab have been associated with the better clinical efficacy of those agents [41,42].

Genetic-based treatment in PsA is a promising idea allowing a personalized approach with an optimized outcome and minimized side effects. Several biomarkers were identified, but further research on the role of genetic factors in response to this treatment is required.

## 5. Soluble Biomarkers of Psoriasis Arthritis Development

Many studies have attempted to identify protein biomarkers that may be potentially useful in detecting cases of psoriasis with a higher risk of joint involvement. Unfortunately, these have been conducted on small cohorts of patients, and the results are often not replicable. A recent meta-analysis by Wirth et al. proposed the potential use of bone and cartilage turnover biomarkers [43]. Several studies have reported an elevated level of serum cartilage oligometrix metalloproteinase (COMP) and serum matrix metalloproteinase-3 (MMP3) in PsA patients in comparison to PsO patients and healthy controls [43]. In addition, in patients with psoriasis, hsCRP (highly sensitive CRP), OPG (osteoprotegerin), and the CPII: C2C ratio (C-propeptide of Type II collagen: collagen fragment neoepitopes Col2-3/4 ratio) were also found to be independently associated with PsA [44]. A higher concentration of IL-18 has also been noted in PsA patients compared to healthy controls, and the authors proposed that IL-18 and IL-20, as well as matrix metalloproteinases 1 and 3, may be suitable markers for the severity of psoriatic arthritis [45].

Two panels of biomarkers distinguishing psoriatic arthritis from osteroarthritis were suggested: (a) COMP, resistin, NGF (nerve growth factor), MCP-1 (monocyte chemoattractant protein-1) [44] and (b) COMP, aggrecan [46]. In addition, Leitjten et al. found an increased serum concentration of tissue-resident memory CD8+ T cells (TRMs) among patients with PsA and suggested that the disturbance of skin homeostasis may contribute to PsA development [47]. A similar conclusion was drawn from another research, which revealed an increased level of IL-17A-producing TRMs in the skin of patients with PsO, and the level of TRMs was correlated with the severity of psoriatic plaque [48]. Moreover, these cells were found in abundance in biopsy specimens taken from psoriatic skin lesions not only in their active phase but also after clinical resolution [49]. Since TRMs might originate from the epidermis, they may play a significant role in psoriasis development. Their presence could explain a disease memory in psoriasis and recurrences in the previously affected areas. However, further research is needed to evaluate the full potential of these indicators.

## 6. Imaging Techniques

Several imaging techniques may be potentially useful in the evaluation of joint involvement. Conventional radiography allows the detection of late stages of the disease, which are characterized by bone erosions and advanced soft tissue changes, often leaving early lesions unrevealed [50]. Bone scintigraphy can be used to detect increased bone metabolism in various inflammatory conditions; however, it is rarely used in clinical practice due to its high sensitivity but relatively low specificity [50]. It is also rarely used in research. Notably, Raza et al. successfully used bone scintigraphy to detect subclinical joint involvement in PsO patients and its response to methotrexate treatment [51].

High-resolution computed tomography (CT) can detect osteoarticular lesions much earlier than conventional radiography and is particularly useful for assessing axial skeleton joints, erosive and proliferative bone lesions and soft tissue calcifications [48]. CT scanning found structural entheseal lesions and low cortical volumetric bone mineral density at entheseal and intraarticular sites to be associated with an increased risk of developing PsA in patients with psoriasis [52].

High-resolution magnetic resonance imaging (MRI) is commonly used for synovial membrane and sacroiliac joint assessment; however, its use in everyday practice is limited by availability and costs [50]. Faustini et al. report the likelihood of developing PsA in patients with symptoms of arthralgia and synovitis detected by MRI to be 55.5% within a year [53].

Lastly, ultrasound (US) is an excellent modality for detecting pathophysiological changes in arthritis due to its effectiveness in identifying early lesions, availability and the rapid assessment of multiple joints. Ultrasound imaging with the greyscale and power Doppler mode can be widely used in the assessment of joints and for monitoring the effectiveness of anti-inflammatory treatment. A greyscale examination allows the visualization of joint effusion, synovial hypertrophy and ligaments, as well as tendons with their sheaths and attachments, while Doppler mode can also be used to identify increased blood flow in the inflamed tissues [50].

Enthesitis is regarded as a hallmark of PsA, and most prevalent entheseal changes include a lack of regular fibrillar architecture, hypoechogenicity, the thickening of tendon insertion, the Doppler activity of tendons, ligaments, and joint capsules at their bony attachment, calcifications and enthesophytes [54,55]. Both enthesitis and synovitis were more prevalent among asymptomatic psoriasis patients compared to among healthy controls; in addition, while PASI appears to be correlated with active enthesitis, a direct association has also been found between baseline US enthesopathy and the risk of PsA development [56,57]. In a two-year follow-up of 109 psoriasis patients of Saudi Arabian origin, the presence of enthesitis, synovitis and CRP level was positively correlated with PsA development [58].

Almost 50% of the healthy population possess detectable subclinical synovitis in at least one joint; however, power Doppler signals can be absent or produce a lower score than in synovia of patients with PsA [56,59]. Therefore, some authors recommend differentiating synovitis (hypertrophy of the synovium) from active synovitis (synovial hypertrophy with increased PD signal) [56]. Moreover, the incidence of tenosynovitis is almost six times higher in patients with non-specific arthralgia than in asymptomatic psoriasis patients, and in most cases, the flexor tendons of the hands are inflamed [57]. The UPSTREAM study group found the Achilles tendon and bursa, flexor and extensor tendons of the digits and proximal interphalangeal joints of the hands to be the most relevant indicators of PsA development [60]. Shear-wave elastography can also be potentially valuable in assessing Achilles tendinopathy [61].

Nail apparatus ultrasonography offers promise in the evaluation of subclinical changes in nail structures, enthesopathies and treatment outcomes. Unfortunately, this technique is not yet standardized and validated, and the data on its efficacy in treatment monitoring is insufficient [62].

Generally speaking, although a number of diagnostic tools are available for assessing psoriasis and psoriatic arthritis, all offer different degrees of sensitivity, specificity, accessibility and price. The choice of imaging method depends on the indications and resources. Joint ultrasound offers advantages over other imaging techniques for the early identification of PsA-related pathologies. Although it is a promising diagnostic and monitoring tool, further research is needed to determine the key imaging features.

## 7. Psoriatic Arthritis Screening Models

Patients with psoriasis should be routinely screened for psoriatic arthritis using the available protocols. Urruticoechea-Arana et al. reviewed twelve questionnaires and recommended the use of the PURE-4 (Psoriatic Arthritis UnclutteRed screening Evaluation) scale in busy clinics [63]. The PURE-4 is based on the following criteria: signs of dactylitis, inflammatory heel pain, bilateral buttock pain and peripheral joint pain with swelling in patients aged <50. Each item is equally weighted (0–1); therefore, the total score is 0–4. Assuming a threshold of ≥1 point, the PURE-4 offers 85.7% sensitivity in PsA detection and 83.6% specificity. The key advantages of the questionnaire are its uncomplicated formula, time efficiency and easy implementation without any preceding training [64] (Table 1).

A combination of protocols, e.g., the PEST/CASPAR screening algorithm, is also recommended [65]. The DAPPER (Discovery of Arthritis in Psoriasis Patients for Early Rheumatological Referral) research group proposes the use of the following: tender joint, swollen joint and dactylitis count, Leeds Enthesitis Index, SPARCC enthesis score and the inflammatory back pain criteria of the Assessment of Spondyloarthritis International Society. In a study of 304 patients in a dermatological outpatient clinic, this approach allowed the detection of five patients with active PsA who did not have a history of PsA and two active PsA patients previously diagnosed with PsA who were not under treatment by a rheumatologist at the time [66,67]. These results confirm the need to improve current psoriatic arthritis screening strategies.

## 8. Early Intervention-Prevention in PsA

The early intervention hypothesis implies that psoriatic arthritis development could be delayed or even prevented if pharmacological intervention is applied at an early window of opportunity. This has been examined in several studies (Table 2).

In a retrospective study of 464 patients with moderate-to-severe plaque psoriasis, the annual incidence rate of PsA was found to be 2.17 cases per 100 patients/year among patients treated with narrow-band ultraviolet light B phototherapy compared to 1.2 cases per 100 patients/year for a group taking biological disease-modifying antirheumatic drugs (bDMARDs) [68]. These results were partially replicated in another study, in which the risk of developing PsA was comparable between patients receiving bDMARDs or cDMARDs (conventional disease-modifying anti-rheumatic drugs); in addition, this risk was significantly lower than that found for other treatment methods (topical, phototherapy or no treatment) [69]. Other findings also indicate a reduced risk of PsA development among patients receiving biological treatment [70,71]. Ultrasonographic enthesitis (thickness and hypoechogenicity of entheses) was found to be reversible after just six months of systemic treatment [72].

The evaluation of novel biological drugs also supports an early intervention hypothesis. In the IVEPSA study, secukinumab (Il-17A inhibition) was administered in PsO patients with a high risk of developing PsA (nail or head psoriasis or PASI > 6) and arthralgia. After 24 weeks of treatment, skin lesions improved, as did the arthralgia and synovitis scores assessed by CT and MRI [73]. In 23 patients with moderate-to-severe plaque psoriasis, ustekinumab (Il-12/IL-23 inhibitor) reduced peripheral subclinical enthesopathy within 12 weeks, and this effect was maintained until the end of the 52-week observation [74]. The efficacy of guselkumab (IL-23 inhibitor) is currently under assessment in an ongoing randomized, double-blind, placebo-controlled multicenter PAMPA trial [75].

However, in contrast, a retrospective cohort study of 193,709 patients with PsO without PsA by Meer et al. found that bDMARD use was associated with the development of PsA. The authors stress that these findings could be confounded by protophatic bias, i.e., the treatment might have been applied for the first symptoms of subclinical and undiagnosed arthritis, leading to the conclusion that the treatment introduction caused the disease [76].

Early intervention is based on the premise that the early administration of systemic treatment may alter the natural course of psoriasis by averting tissue memory consisting of molecular and cellular changes, inter alia TRMs [77]. The concept was validated for other immune-mediated diseases, such as Crohn’s disease [78] and rheumatoid arthritis [79]. This idea is particularly appealing since, hypothetically, it delays or prevents PsA and thus provides better long-term outcomes, potentially lowering the risk of complications, such as cardiovascular events, metabolic syndrome and disability. However, patients receiving systemic treatment often require scheduled control visits and frequent laboratory monitoring and are at risk of adverse drug effects and drug toxicity; in addition, early intervention does incur financial costs, which should also be included in further research [80]. Next, it is necessary to deduce whether the treatment modality is of lesser importance when preventing PsA development as long as the patient receives systemic treatment. In the authors’ view, prospective studies should be performed, comparing the potential of cDMARDs versus bDMARDs to prevent the development of PsA.

Finally, although biological agents are often discussed as a class, in fact, they constitute a diverse group, which can be subdivided according to the target molecules, biochemical structure, and pharmacokinetic or pharmacodynamic properties. In terms of bDMARDs in psoriasis treatment, they are usually divided into targeting cells (T-cells or antigen-presenting cells) and cytokines (for example, TNF (tumor necrosis factor), IL-12, IL-17, IL-23). Further intra-class subdivision is possible based on the clinical profiles of these agents. For instance, in the anti-TNF group, substances could vary in structure (etanercept is a fusion protein, infliximab is a chimeric monoclonal antibody, adalimumab is a fully human antibody), the binding site—the specificity and affinity of binding, plasma concentration behaviors (steady concentration with etanercept versus peaks with infliximab administration), dosing schedules and routes of administration and adverse events [81]. It is important to acknowledge those differences before prescribing the treatment to a particular patient. Therapy with biological agents should be personalized and tailored to the individual in order to select the most effective strategy. Perhaps pharmacogenetic biomarkers could facilitate matching the characteristics of the drug with the unique profile of the patient.

To conclude, initial data on early intervention efficacy is promising, but more evidence is needed before its routine implementation in the healthcare system.

**Table 2:** Studies investigating the impact of treatment on the development of psoriatic arthritis in psoriasis patients.

Study	Study Design	No of Patients	Treatment	Findings
Gisondi et al. [68]	Retrospective non-randomized study	464	NB-UVB phototherapy vs. bDMARDs (TNFi, IL-17i, IL-12/23i)	Lower risk of PsA development in patients on bDMARDs compared with phototherapy
Acosta Felquer et al. [69]	Retrospective cohort study	1719	Topicals (topical treatment, phototherapy, no treatment) vs. cDMARDs (MTX, CsA) vs. bDMARDs (TNFi, IL-17i, IL-12/23i).	Lower risk of PsA development in patients on bDMARDs compared with topicals but not compared with cDMARDs
Rosenthal et al. [70]	Retrospective cohort study	1326	bDMARDs (TNFi, IL-12/23i, IL-17i, IL/23i) vs. other treatment	Lower risk of PsA development in patients on bDMARDs
Solmaz et al. [71]	Retrospective chart review	203	bDMARDs (TNFi, IL-17i, IL-12/23i) vs. cDMARDs vs. other/no treatment	Lower risk of PsA development in patients on bDMARDs and cDMARDs
Acquacalda et al. [72]	Prospective study	34	cDMARDs (MTX, CsA, Aci) vs. bDMARDs (TNFi, IL-12/23i)	cDMARDs and bDMARDs improve enthesitis
Kampylafka et al. [73]	Prospective study	20	Secukinumab (IL-17i)	Secukinumab improves subclinical inflammatory joint changes
Savage et al. [74]	Prospective study	23	Ustekinumab (IL-12/23i)	Ustekinumab improves subclinical enthesopathy
Haberman et al. [75]	Randomized, double-blind, placebo-controlled, trial	200	Guselkumab (IL-23i) vs. placebo	Ongoing study
Meer et al. [76]	Retrospective cohort study	193,709	bDMARDs (TNFi, IL-17i, IL-12/23i, IL-23i) vs. cDMARDs/phototherapy	Higher risk of PsA development in patients on bDMARDs compared with cDMARDs/phototherapy
Napolitano et al. [82]	Retrospective study	434	bDMARDs (TNFi, IL-12/23i)	In some patients bDMARDs may not prevent PsA development
Balato et al. [83]	Retrospective study	326	phototherapy	Phototherapy is not able to prevent or delay PsA development

Abbreviations: NB-UVB—narrow-band ultraviolet B, bDMARDs—biological disease-modifying antirheumatic drugs, cDMARDs—conventional disease-modifying antirheumatic drugs, TNFi—TNF inhibitors, IL-17i—IL-17 inhibitors, IL-12/23i—IL-12/23 inhibitors, IL-23i—IL-23 inhibitors, PsA—psoriatic arthritis, MTX—methotrexate, Csa—ciclosporin A, Aci—acitretin.

## 9. Summary and Conclusions

Several risk factors predispose psoriasis patients to arthritis development. Dermatologists play a key role in detecting early changes associated with PsA in high-risk patients, and screening for PsA should be routinely performed in cases of psoriasis. Early systemic treatment may have a significant positive impact on the long-term outcomes of psoriasis by delaying or preventing joint involvement. A treat-to-target strategy for preventing PsA development is worth further study.

## Figures and Tables

**Table 1:** PURE-4 questionnaire [64].

Symptoms	Questions
Evocative signs of dactylitis	Have you ever had a globally swollen and painful finger or toe?
Inflammatory heel pain	Have you ever had heel pain as soon as you stand up in the morning?
Bilateral buttock pain	Have you ever had left and right buttock pain, at the same time or not?
If age > 50 years, peripheral joint pain with swelling	Have you ever had a swollen and painful joint?

0–1 point for each item. Total score 0–4 points. For ≥ 1 point, sensitivity = 85.7%, specificity = 83.6% for detecting psoriatic arthritis.
